# “Texas-Sized” Molecular Boxes: From Chemistry to Applications

**DOI:** 10.3390/molecules26092426

**Published:** 2021-04-21

**Authors:** Xiaodong Chi, Jinya Tian, Dan Luo, Han-Yuan Gong, Feihe Huang, Jonathan L. Sessler

**Affiliations:** 1 State Key Laboratory of Materials Processing and Die & Mould Technology, School of Materials Science and Engineering, Huazhong University of Science and Technology, Wuhan 430074, China; tianjinya@hust.edu.cn (J.T.); dluo@hust.edu.cn (D.L.); 2 College of Chemistry, Beijing Normal University, No. 19, Xinwai Street, Beijing 100875, China; 3 State Key Laboratory of Chemical Engineering, Key Laboratory of Excited-State Materials of Zhejiang Province, Stoddart Institute of Molecular Science, Department of Chemistry, Zhejiang University, Hangzhou 310027, China; 4 Department of Chemistry, The University of Texas at Austin, Austin, TX 78712-1224, USA

**Keywords:** supramolecular chemistry, macrocyclic compounds, self-assembly, host–guest chemistry, supramolecular polymers, anion recognition, hydrogel

## Abstract

The design and synthesis of novel macrocyclic host molecules continues to attract attention because such species play important roles in supramolecular chemistry. However, the discovery of new classes of macrocycles presents a considerable challenge due to the need to embody by design effective molecular recognition features, as well as ideally the development of synthetic routes that permit further functionalization. In 2010, we reported a new class of macrocyclic hosts: a set of tetracationic imidazolium macrocycles, which we termed “Texas-sized” molecular boxes (TxSBs) in homage to Stoddart’s classic “blue box” (CBPQT^4+^). Compared with the rigid blue box, the first generation TxSB displayed considerably greater conformational flexibility and a relatively large central cavity, making it a good host for a variety of electron-rich guests. In this review, we provide a comprehensive summary of TxSB chemistry, detailing our recent progress in the area of anion-responsive supramolecular self-assembly and applications of the underlying chemistry to water purification, information storage, and controlled drug release. Our objective is to provide not only a review of the fundamental findings, but also to outline future research directions where TxSBs and their constructs may have a role to play.

## 1. Introduction

Macrocyclic compounds and hosts, combining molecular recognition and complexation properties, have played a significant role in the development of supramolecular chemistry [1,2,3,4,5,6]. Renowned supramolecular hosts, such as crown ethers [7,8], cyclodextrins (CDs) [9,10], Stoddart’s “blue box” [11], calix[n]arenes [12,13], cucurbit[n]urils (CBs) [14,15], and pillar[n]arene [2,16] have been widely explored as building blocks for host–guest molecular recognition [17,18], energy storage [19], intelligent materials [20,21,22,23], drug delivery systems [24,25,26,27], and molecular machines [28,29,30], etc. A number of new synthetic macrocycles have been reported and have received considerable attention, such as Wang’s corona[n]arenes [31], Jiang’s oxatub[4]arene [32], Flood’s cyanostar [33], and others [34]. Among the cationic macrocycles, the tetracationic cyclobis(paraquat-*p*-phenylene) (CBPQT^4+^), also known as the “blue box” [11] and structurally related macrocycles developed by Stoddart and co-workers, are probably the most iconic. To date, a variety of supramolecular structures have been prepared that exploit the rigid box-like geometry of CBPQT^4+^ and the fact that it promotes strong donor–acceptor interactions with various electron-rich species; many of the constructs prepared from CBPQT^4+^ have shown potential applications in fields as diverse as molecular recognition, self-assembly [35], molecular electronics [36], and molecular machines [37].

In 2010, we prepared a first generation imidazolium-based macrocycle [38], which we termed the “Texas-sized” molecular box (TxSB) in homage to Stoddart’s classic “blue box” (CBPQT^4+^). Compared to the latter relatively rigid structure, the TxSB was characterized by a larger central cavity and greater conformational flexibility. These features and the cationic, electron-deficient nature of the system made the TxSB attractive as a host for various electron-rich guests and as a building block for supramolecular self-assembly, including the fabrication of (pseudo)rotaxanes or poly(pseudo)rotaxanes, supramolecular dimers and polymers [39], as well as other functional materials [40]. Herein we summarize recent advances in the synthesis of TxSBs, their host–guest binding properties, anion-induced self-assembly, and applications of TxSB-based materials to address challenges in the areas of water purification, information storage, and controlled drug release.

## 2. Synthesis of “Texas-Sized” Molecular Box

**Scheme 1:**
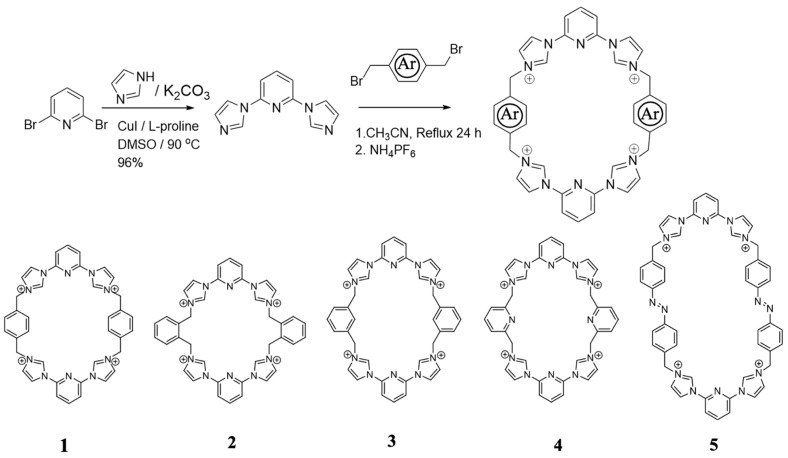
Syntheses and structures of the tetracationic “Texas-sized” molecular box (TxSB) **1** and analogues **2**–**5**.

Roughly a decade ago and inspired by the previous progress in the field of imidazolium-based anion recognition [41], we considered that it would be possible to develop new tetracationic imidazolium macrocycles, which would act as larger analogues of the blue box developed by Stoddart and coworkers [42]. To address this challenge, the bis-imidazole precursor shown in Scheme 1 was synthesized from 2,6-dibromopyridine in high yield (96%) via an Ullmann-type coupling reaction. It was then cyclized with 1,4-bis(bromomethyl)benzene to yield the tetrabromide salt. Upon the addition of aqueous ammonium hexafluorophosphate, a precipitate formed, which was filtered off to yield the corresponding tetrahexafluorophoshate salt, receptor **1**, in 58% yield (over two steps) [38]. Using a similar synthetic strategy, the bridged aromatic subunit used to prepare **1**, namely, 1,4-bis(bromomethyl)benzene, could be replaced by other bridge-providing precursors, such as 1,2-bis(bromomethyl)benzene [43], 1,3-bis(bromomethyl)benzene [43], 1,3-bis(bromomethyl)-pyridine [43], and 1,4-bis(bromomethyl)azobenzene [44]. This allowed preparation of the new tetracationic imidazolium macrocycles **2**, **3**, **4**, and **5**, respectively (Scheme 1).

## 3. Host–Guest Chemistry of “Texas-Sized” Molecular Box

The pursuit of host–guest recognition has been a major driver for the development of macrocyclic chemistry in recent decades, as have been opportunities involving the construction of functional self-assembled systems [45,46]. The Texas-sized boxes of Scheme 1 constitute a new generation of macrocycles endowed with interesting charge and conformational features. Not surprisingly, therefore, considerable effort has been devoted to investigating their fundamental host–guest properties.

TxSB **1** was found capable of attracting and enclosing a variety of electron-rich neutral and anionic guests. This was attributed to its structure, size, flexibility, and the presence of both cationic imidazolium moieties and neutral aromatic subunits within the ring. Taking these features into account, early on various carboxylate anions were used to explore the host–guest interaction behavior of **1** [47,48,49].

**Figure 1:**
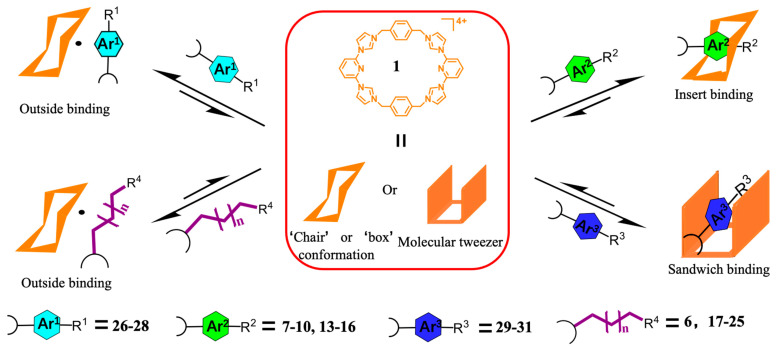
Schematic representations of the limiting binding modes observed when the macrocycle **1** is treated with carboxylate anionic precursors containing different aromatic rings. The associated anion recognition details are summarized in Table 1. Note that the B the location-defining terms of “Outside Cavity”, “Threaded through Cavity”, and “Sandwich” in Table 1 correspond to “Outside binding”, “Insert binding”, and “Sandwich binding” in this figure, respectively. This image is a modification of one that first appeared in ref. [50] and is reproduced with permission. Copyright 2015 The Royal Society of Chemistry.

We summarize the host–guest binding modes inferred for the complexes formed between **1** and different anionic carboxylate species in Table 1, and these modes are shown schematically in Figure 1. It was found that most aromatic anions with “widths” less than 5.8 Å thread through the cavity of macrocycle **1** to form pseudorotaxanes. For ease of reference, this was termed the “insert” binding mode (Figure 1) [38,47,48,50]. Meanwhile, in the case of larger conjugated aromatic anions, it was found that TxSB **1** can serve as “molecular tweezers”, giving rise to sandwich-like complexes. In contrast, linear aliphatic anions and aromatic anions with “widths” larger than 5.8 Å bind preferentially to the exterior of **1** (Figure 1). 

**Figure 2:**
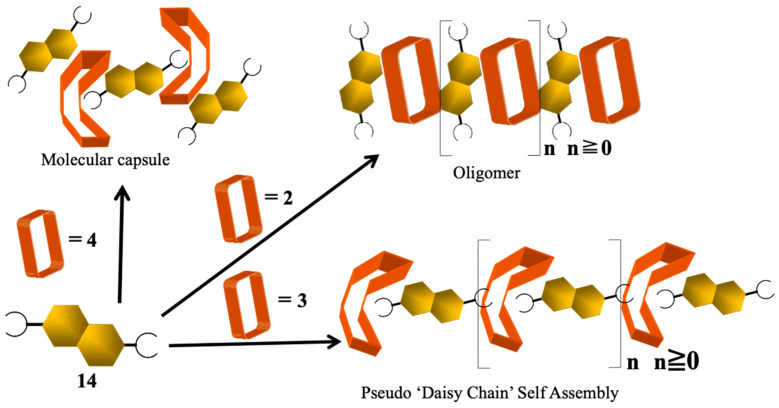
Cartoon representations of interlocked ensembles supported by the congeneric macrocycles **2**‒**4** in conjunction with 2,6-naphthalenedicarboxylate dianion. Modified from ref. [43]. Copyright 2013 American Chemical Society.

We also studied how the bridges (derived from the bis(bromomethyl)arenes) affected the host–guest interactions. In this context, the congeneric macrocycles **2**‒**4** were studied. Briefly, it was found that this set of receptors permitted access to a wide range of self-associated ensembles using the 2,6-naphthalenedicarboxylate dianion in all cases and under otherwise analogous conditions (Figure 2). The diversity of the structures obtained led to the conclusion that the bridges between two imidazolium moieties in these macrocycles play an important role in defining the host–guest interactions [43].

**Figure 3:**
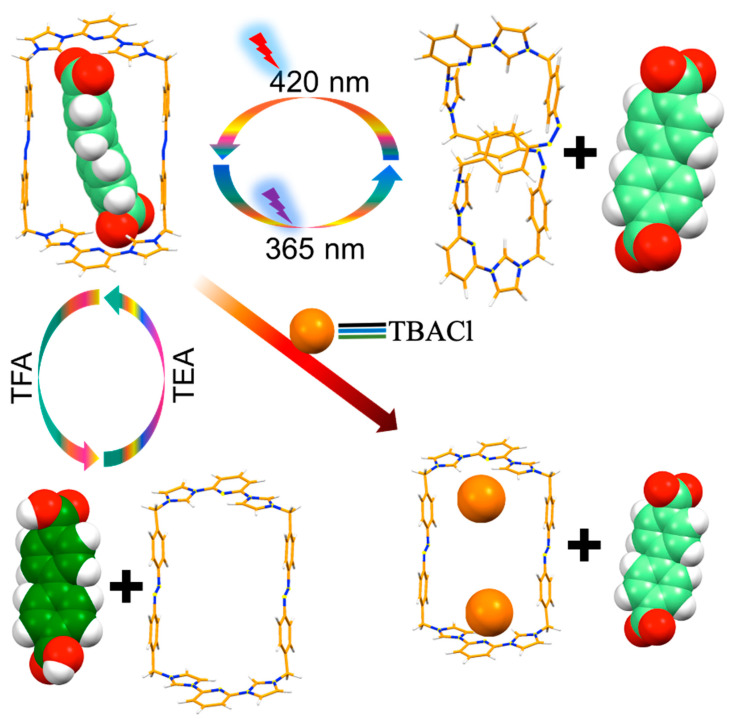
Cartoon representation of the multi-responsive capture-and-release of guest by TxSB **5**, including photo-, pH-, and anion-responsiveness. Reproduced from ref. [44]. Copyright 2019 American Chemical Society.

Functionalized macrocyclic receptors are necessary for many molecular recognition- and self-assembly-related applications. “Texas-sized” molecular boxes containing functional groups, such as azobenzene, or bearing carboxylic acid-substituents TxSB were thus synthesized. By way of example, we reported an expanded “Texas-sized” molecular box (**5**), which was prepared by introducing azobenzene group into the macrocyclic framework [44]. In this instance, the shape of the receptor cavity could be controlled through photoirradiation. In the absence of UV light, this particular TxSB exists in the *E*,*E*-configuration, which was found to encapsulate effectively aryl anions in DMSO-*d*_6_. Upon exposure to UV light (365 nm), the cavity shape changed as the result of E --> Z configurational switching of the azobenzene groups, which leads to the release of the guests from **5** (Figure 3). The use of visible light (420 nm) allowed the system to be reset back to the previous state. In addition, it was also found that the capture and release of guest molecules could be controlled by chemical competition and or by modulating the pH.

## 4. Self-Assembled Ensembles Containing a “Texas-Sized” Molecular Box

**Figure 4:**
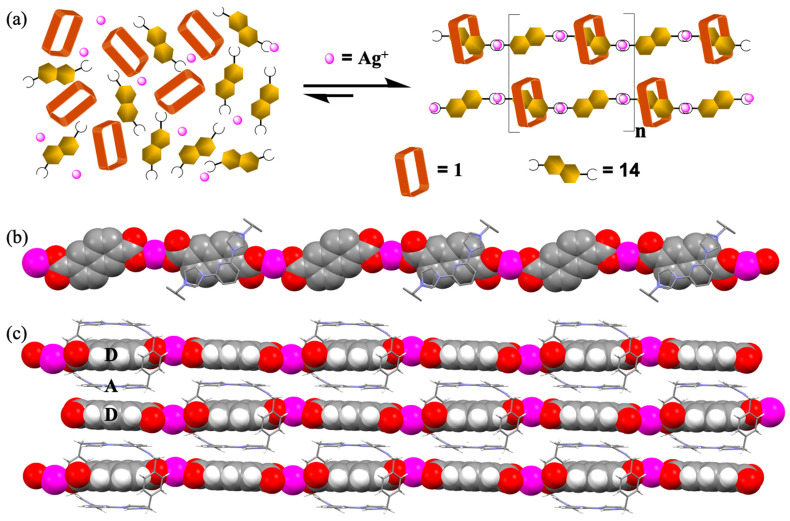
(**a**) Assembly of a silver-linked polypseudorotaxane constructed from TxSB **1**, Ag(I), and the 2,6-naphthalenedicarboxylate dianion. (**b**) Top and (**c**) side views of the silver-linked polypseudorotaxane chain seen in the solid state. A and D stand for acceptor and donor, respectively. Note: Compound numbers are provided in Table 1. Modified with permission from a figure that first appeared in ref. [51]. Copyright 2011 American Chemical Society.

**Figure 5:**
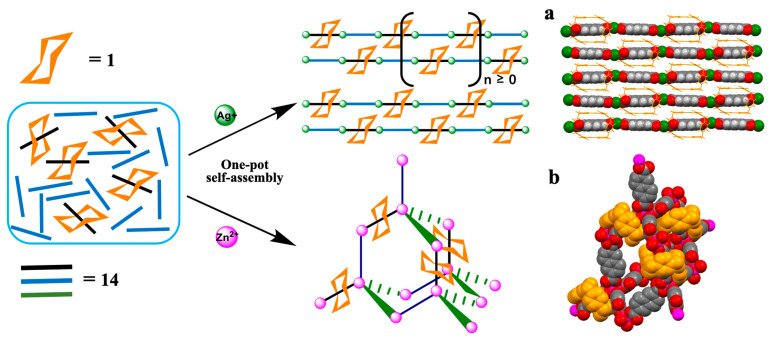
Schematic representations and structures of the 2D array made up of 1D polyrotaxane subunits and the 3D metal–organic rotaxane frameworks (MORFs) formed from the TxSB 1 and the 2,6-naphthalene dicarboxylic dianion. Note: Compound numbers are provided in Table 1. Modified with permission from a figure that first appeared in ref. [52]. Copyright 2011 The Royal Society of Chemistry.

As discussed above, the TxSB can interact with various anions, such as carboxylate anions, to produce a variety of self-assembled ensembles. Carboxylate anions are widely used ligands to construct functional self-assembled ensembles, such as metal organic frameworks (MOFs), in the presence of various metal cations. In order to access higher order, multi-component self-assembled contructs, Ag^+^ was first tested as the cation, which can form roughly linear 1:2 (cation:anion) complexes with carboxylate anions [51]. An insoluble precipitate formed when Ag^+^ was added to a solution of **1** containing 1 molar equiv of 2,6-naphthalene dicarboxylic acid and 5 molar equiv of TEA. Based on a single crystal structural analysis, this precipitate was found to consist of a 1D supramolecular polymer. The structure itself contains monomeric units with the individual polyrotaxane chains being arranged in an ordered two-dimensional DAD array (Figure 4). It was found that the formation, degree of aggregation, and the nature of the complexes themselves could be easily influenced by the added guests, a finding that was ascribed in part to the flexible of the TxSB framework. When the cation was changed to Zn^2+^, for instance, a multicomponent self-assembled interlocked 3D metal–organic rotaxane framework (MORF) was formed (Figure 5) [52].

**Figure 6:**
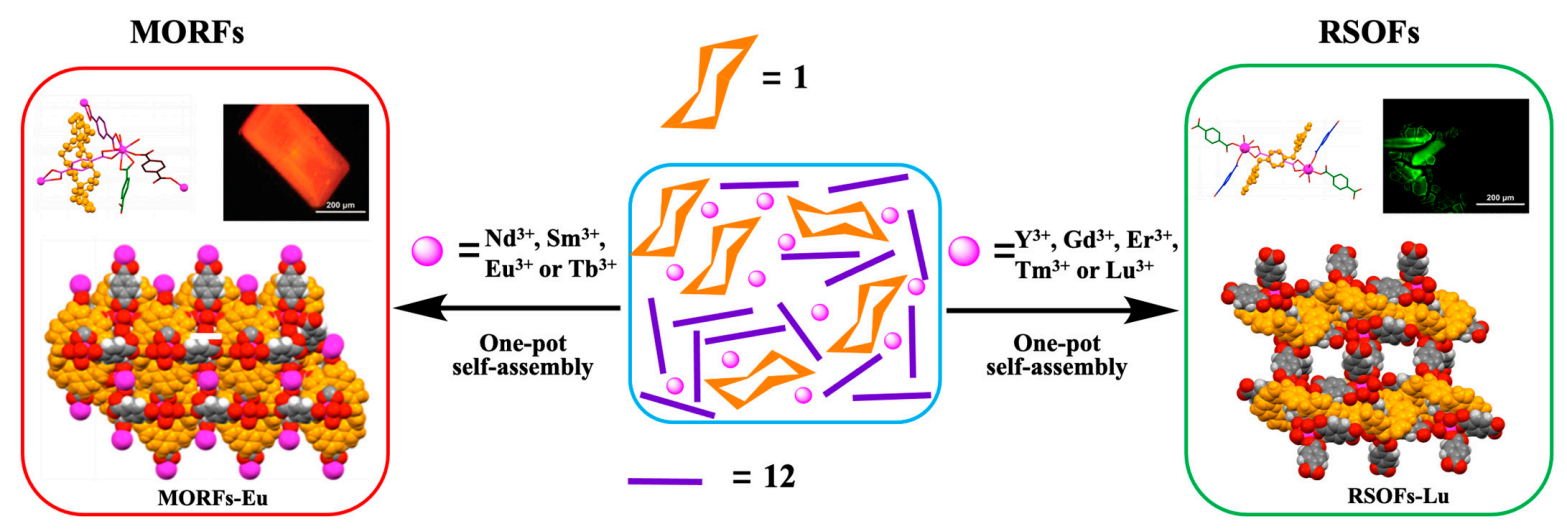
One-pot self-assembly of 3D metal–organic rotaxane frameworks (MORFs and rotaxanated supramolecular organic frameworks (RSOFs) containing the TxSB **1**, the terephthalate dianion and various lanthanide(III) metal cations. Note: Compound numbers are provided in Table 1. Modified with permission from a figure that first appeared in ref. [53]. Copyright 2012 The Royal Society of Chemistry.

In order to study the metal cations dependence on the supramolecular self-assembly involving TxSB 1 and anions, the terephthalate dianion was investigated, in combination with trivalent lanthanide cations [53]. In the absence of these cations, the terephthalate dianion favors an “outside” binding mode in interacting with 1. However, selected trivalent lanthanide metal cations, in particular, Nd^3+^, Sm^3+^, Eu^3+^, and Tb^3+^, were found to induce the terephthalate dianion to insert into the cavity of macrocycle 1 to form MORFs (Figure 6). In contrast, several other lanthanide metal cations, e.g., Y^3+^, Gd^3+^, Er^3+^, Tm^3+^, and Lu^3+^, were found to interact with 1 and the terephthalate dianion to form unusual rotaxanated supramolecular organic frameworks (RSOFs).

**Figure 7:**
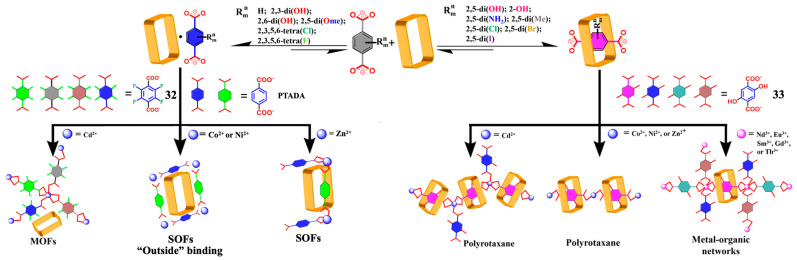
Schematic showing how the substituents defining a particular *p*-terephthalic acid dianion (PTADA) serve to stabilize various supramolecular constructs via interaction with the cationic macrocycle **1**. Modified with permission from a figure that first appeared in ref. [54]. Copyright 2020 American Chemical Society.

Substituent effects always play critical roles in regulating chemical reactions, as well as self-assembled structures. Appreciating this, we explored how different substituents on various putative anionic guests would affect their interactions with the TxSB **1** [54]. Monosubstituted or *para*-disubstituted derivatives on the terephthalate dianion favor formation of pseudorotaxane structures when allowed to interact with **1**. In contrast, the *p*-terephthalic acid dianion (PTADA), and its derivatives (2,3-di(OH PTADA), 2,6-di(OH PTADA) and 2,3,5,6-tetra(Cl) PTADA), lead to complexes where the substrate is bound to the outside of **1** (Figure 7). The different binding modes stabilized by the various PTADA derivative were, in turn, found to modulate further supramolecular self-assembly when the self-assembly process was carried out in the presence of selected metal cations (M). In fact, a number of self-assembled ensembles could be constructed, including one-dimensional polyrotaxanes, outside-type rotaxanated supramolecular organic frameworks (RSOFs), and two-dimensional metal−organic rotaxane frameworks (MORFs), depending on the particular choice of PTADA derivative and metal cation (M = Co^2+^, Ni^2+,^ Zn^2+^, Cd^2+^, Gd^3+^, Nd^3+^, Eu^3+^, Sm^3+^, Tb^3+^).

**Figure 8:**
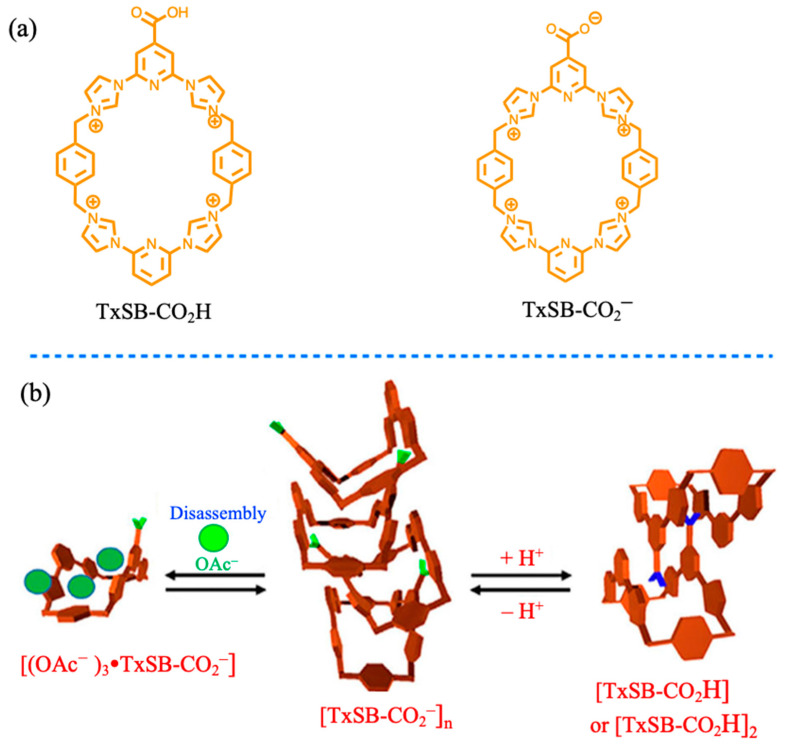
(**a**) Chemical structures of TxSB-CO_2_H and its corresponding base TxSB-COO^−^. (**b**) Schematic view of self-assembly of a supramolecular polymer constructed from mono functionalized TxSB-CO^2–^, which proved both anion and pH responsive (see text for details). Reproduced from ref. [39]. Copyright 2018 American Chemical Society.

In an effort to explore the potential usability of TxSBs in the context of materials science, we recently designed and synthesized a mono-carboxylic acid-functionalized “Texas-sized” molecular box [39]. This new derivative can self-associate to form an extended array of dimers in the solid state, as determined via a single crystal X-ray diffraction analysis. However, when TxSB-CO_2_H was converted to its conjugate base TxSB-CO^2−^ by treatment with TEA, the resulting anion-containing monomers self-associate into higher-order head-to-tail oligomers [TxSB-CO^2−^]_n_ in solution and in the solid state (Figure 8). The ensembles produced from TxSB-CO^2−^ proved environmentally responsive, with the extent of assembly being readily modulated via changes in the temperature, pH, and concentration, as well as via the addition of the acetate anion, which acted as a competitive guest.

## 5. Applications of “Texas-Sized” Molecular Box

Soft materials have received considerable attention from chemists and material scientists due to the advantages of their soft, flexible nature and the convenience of the preparation process. Soft materials with anion recognition features could lead to new macromolecular systems of interest in the context of numerous applications. In this section, we discuss application of TxSBs in the fields of water purification, information storage, and controlled drug release.

### 5.1. Hydrogel for Removal of Anions from Water

**Figure 9:**
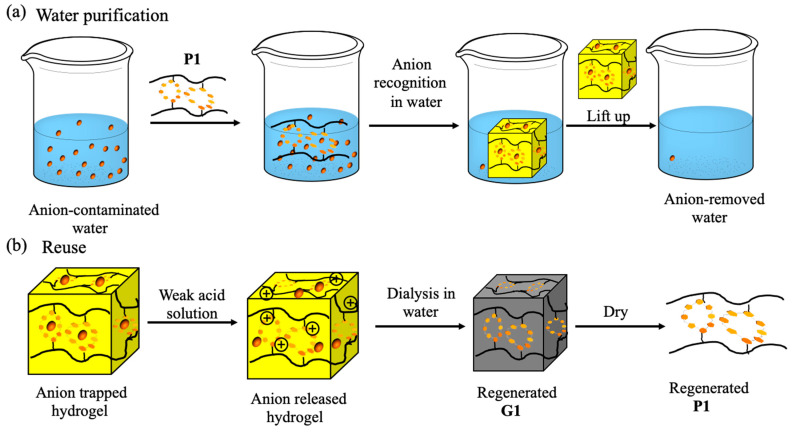
(**a**) Anion removal from a simulated contaminated aqueous source phase by use of a polymeric network P1 containing a TxSB derivative that serves as a tetracationic macrocyclic anion receptor. (**b**) Regeneration of P1 by means of dialysis to give G1. Reproduced from ref. [55]. Copyright 2018 American Chemical Society.

We prepared a hydrogel-forming polymer network, using a water-soluble tetracationic macrocycle **1** as a crosslinker [55]. Compared with traditional anion responsive hydrogels, the hydrogel containing **1** proved capable of recognizing certain anions in aqueous media without disrupting the core hydrogel structure. High anion capture efficiencies were observed. For instance, when this polymer network was immersed in aqueous solutions containing various inorganic and organic salts, absorption of the constituent anions into the polymer network was seen. This led to a change in the physical properties of the hydrogel (Figure 9). The effective absorption seen for this system was ascribed to host−guest interactions between the macrocyclic host and anions. Removal of the anions from the mock contaminated environment could be easily achieved via lifting the guest-containing hydrogels out of the aqueous phase. The hydrogel could then be regenerated for further use by adding dilute HCl, which led to the release of the bound anions. The TxSB-containing polymer network was thus found to define a potentially attractive approach to removing undesired anions from aqueous environments.

### 5.2. Multifluorescent Hydrogels for Encoding, Reading, and Transforming Information

**Figure 10:**
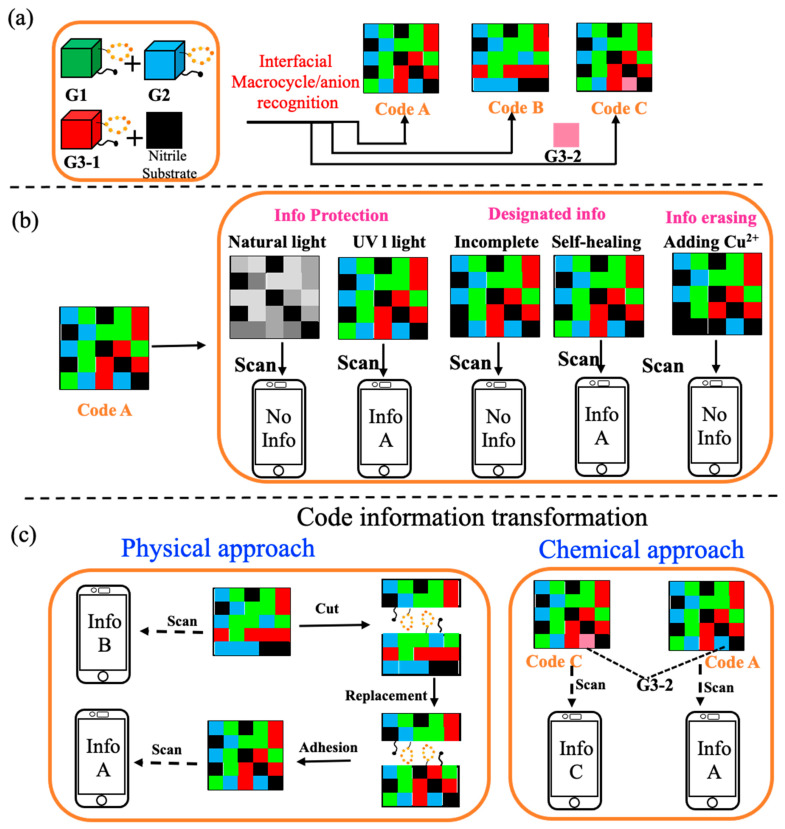
(**a**) Cartoon representation of construction of 3D color codes through the interfacial adhesion of various colored hydrogels. (**b**) Code used for information protection, designated information, and information erasing. (**c**) Schematic view showing how initially encrypted information could be transformed by physical and chemical means. Reproduced with permission from ref. [56]. Copyright 2018 Wiley-VCH.

Recently, we described the creation of information code arrays predicated on anion-recognition-based gels. A series of hydrogels were achieved via incorporating a poly(acrylamide) (pAAm) network cross-linked by *N*,*N*′-methylenebis(acrylamide) (MBAAm) containing both TxSB moieties (as their tetrakis-chloride anion salts) and alkylsulfonate subunits [56]. Various fluorescent molecules were introduced separately into the polymer chains of three of the TxSB- and sulfonate-bearing hydrogels (Figure 10a). The fluorescent color 3D codes (Code A, Code B, and Code C) could be easily prepared through interfacial adhesion of different colored fluorescent hydrogels. We could then store encrypted information in the form of the 3D codes, which could be readily read out with a smartphone (Figure 10b). Meanwhile, we could easily transform the encoded information (Code A --> Code B) by switching the hydrogel units with different hydrogel components, which could be realized through a dynamic contact-breaking and readhesion process. Furthermore, we could change the encoded information chemically by introducing a base-responsive fluorophore into a hydrogel array to obtain Code C. When the hydrogel was treated with ammonia, the Code C was converted to Code A quickly (Figure 10c). Therefore, information encrypted by these colored gel arrays could be altered either chemically or physically (Figure 10). This approach is attractive in terms of creating potentially wearable materials coding for specific information. However, this latter promise has yet to be realized.

### 5.3. TxSB-Based Amphiphilic Copolymer for Controlled Release

**Figure 11:**
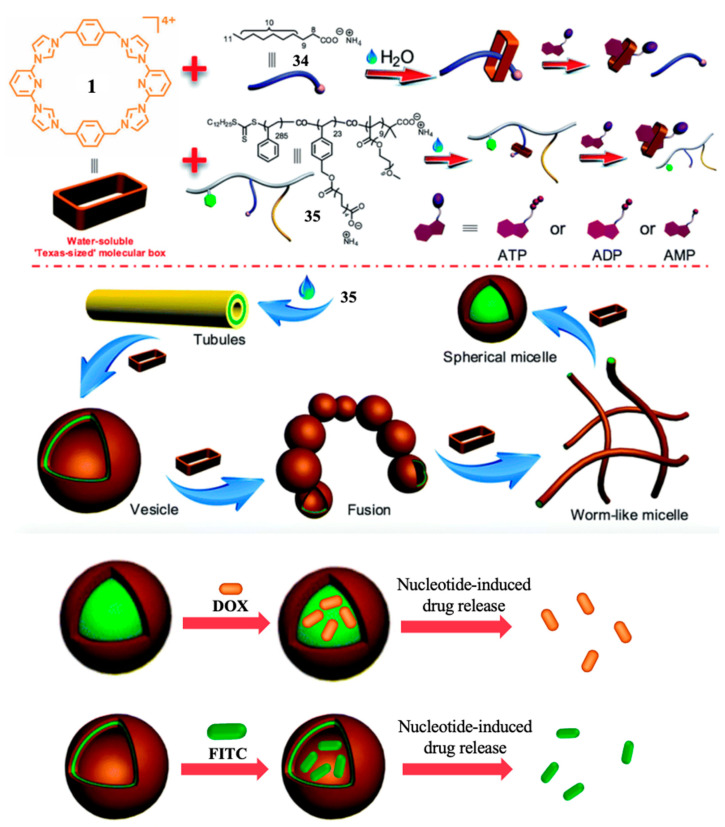
Chemical structures and cartoon representation of nucleotides-responsive host–guest interactions, schematic views of the diverse assemblies formed from **1** and **11** due to the molecular recognition, and nucleotide-induced drug release. Modified with permission from a figure that first appeared in ref. [57]. Copyright 2016 The Royal Society of Chemistry.

Amphiphilic copolymers are of interest because they can stabilize a diverse variety of self-assembled structures in aqueous media. For instance, they can support the formation of inter alia spherical micelles, disklike micelles, cylindrical micelles, vesicles, and other interesting aggregates. We explored the use of TxSB-based anion receptor interactions to stabilize various morphologies. As shown in Figure 11, a supra-amphiphilic polypseudorotaxane could be formed through the host–guest interaction between a water-soluble TxSB **1** (as their tetrakis-chloride anion salts) and an amphiphilic copolymer containing carboxylate anions. The ratio of the hydrophilic part of the supra-amphiphilic polypseudorotaxane could be tuned by adding differing amounts of TxSB **1**. This leads to a change in the overall self-assembled morphology, a feature that could be used to affect controlled drug release [57]. The viability of this strategy was demonstrated by encapsulating the hydrophilic fluorescein isothiocyanate anion (FITC) within vesicles, as well as the hydrophobic chemotherapeutic agent doxorubicin (DOX) within spherical micelles. Nucleotides, such as adenosine-5′-triphosphate (ATP), adenosine-5′-diphosphate (ADP), and adenosine-5′-monophosphate (AMP), can compete with the key host–guest interactions that stabilize these polymer ensembles, making them good candidates to undergo controlled drug release agents. Further study revealed that ATP was the most efficient nucleotide for promoting the controlled release of the encapsulated FITC and DOX. This work was considered to represent a first step towards the study of the “Texas-sized” molecular boxes in the area of stimuli-responsive drug delivery. Given the diversity of structures that may be potentially stabilized and the large number of useful substrates that could likely be delivered, we suggest that this application area warrants further development.

## 6. Summary and Outlook

In summary, we have developed a series of tetracationic imidazolium macrocycles, colloquially known as “Texas-sized” molecular boxes (TxSBs **1**, **2**, **3**, **4,** and **5**). Their ability to recognize organic carboxylate led to the stabilization of a number of monomeric and self-assembled complexes. The binding modes inherent in these complexes and their aggregation features were found to be highly dependent on the choice of macrocycle and anionic substrate. It was also found that other inputs, including protons, competing anions, neutral molecules, and metal cations, could be used to regulate the nature of the structures formed. By judicious choice of the precursors and conditions, a variety of mechanically interlocked molecular architectures including, e.g., metal organic rotaxanes, MORFs, RSOFs, etc., could be obtained. Many of the complexes produced from the “Texas-sized” molecular boxes and the higher order aggregates formed from subsequent self-assembly were found to respond to changes in the environment, such as concentration, pH, temperature, presence of potentially competitive substrates, etc. The various TxSB-based hydrogels produced to date show promise in a number of application areas. For instance, this class of hydrogels has been used to remove surrogate anionic pollutants from aqueous source phases. Related systems were found useful for encoding information in a controllable pattern-based fashion.

On the basis of the work carried out to date, we believe that this versatile molecular receptor will continue to find application in the field of molecular recognition and self-assembly and will permit ultimately the construction of yet more complex molecular switches and externally triggered supramolecular arrays. To date, the “Texas-sized” molecular box and its congeners have allowed for the generation of a number of molecular frameworks with threaded structures. However, this is likely just the beginning. Given the facile synthesis of these macrocycles and the variety of molecular structures that they have been used to stabilize since the first generation system was introduced in 2010, we predict that “Texas-sized” molecular boxes and related cyclic imidazolium macrocycles will continue to attract attention within the self-assembly, anion recognition, cation coordination, and soft materials communities. Efforts to explore new opportunities associated with this class of receptors are currently ongoing in our laboratories.

## Figures and Tables

**Table 1:** Summary of host–guest binding properties of **1** with a wide range of guests in DMSO-*d*_6_ [47,48].

Guest	Binding Site	H:G Binding Ratio	Equilibrium	Association Constant
**6** 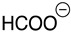	Outside Cavity	2:3	[H] + [G] $\overset{K_{1}}{⇄}$ [H–G] 2[H–G] + [G] $\overset{K_{2}}{⇄}$ [H_2_–G_3_]	*K*_1_ = (8.3 ± 0.4) × 10^3^ M^−1^ *K*_2_ = (2.3 ± 0.1) ×10^6^ M^−2^
**7** 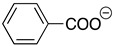	Threaded through Cavity	1:2	[H] + [G] $\overset{K_{1}}{⇄}$ [H–G] [H–G] + [G] $\overset{K_{2}}{⇄}$ [H–G_2_]	*K*_1_ = (1.0 ± 0.1) × 10^4^ M^−1^ *K*_2_ = (3.5 ± 0.2) × 10^3^ M^−1^
**8** 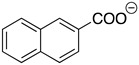	Threaded through Cavity	1:2	[H] + [G] $\overset{K_{1}}{⇄}$ [H–G] [H–G] + [G] $\overset{K_{2}}{⇄}$ [H–G_2_]	*K*_1_ = (8.3 ± 0.4) × 10^3^ M^−1^ *K*_2_ = (2.3 ± 0.2) × 10^3^ M^−1^
**9** 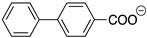	Threaded through Cavity	2:3	[H] + [G] $\overset{K_{1}}{⇄}$ [H–G] 2[H–G] + [G] $\overset{K_{2}}{⇄}$ [H_2_–G_3_]	*K*_1_ = (9.1 ± 0.5) × 10^3^ M^−1^ *K*_2_ = (8.1 ± 0.4) × 10^5^ M^−2^
**10** 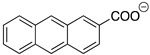	Threaded through Cavity	1:2	[H] + [G] $\overset{K_{1}}{⇄}$ [H–G] [H–G] + [G] $\overset{K_{2}}{⇄}$ [H–G_2_]	*K*_1_ = (6.1 ± 0.3) × 10^2^ M^−1^ *K*_2_ = (7.2 ± 0.4) × 10^2^ M^−1^
**11** 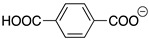	Threaded through Cavity	2:3	[H] + [G] $\overset{K_{1}}{⇄}$ [H–G] 2[H–G] + [G] $\overset{K_{2}}{⇄}$ [H_2_–G_3_]	*K*_1_ = (2.1 ± 0.5) × 10^3^ M^−1^ *K*_2_ = (2.4 ± 0.2) × 10^5^ M^−2^
**12** 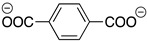	Outside Cavity	1:1	[H] + [G] $\overset{K_{a}}{⇄}$ [H–G]	*K*_a_= (3.3 ± 0.1) × 10^3^ M^−1^
**13** 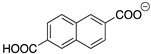	Threaded through Cavity	1:1	[H] + [G] $\overset{K_{a}}{⇄}$ [H–G]	*K*_a_= (2.1 ± 0.1) × 10^3^ M^−1^
**14** 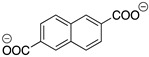	Threaded through Cavity	1:1	[H] + [G] $\overset{K_{a}}{⇄}$ [H–G]	*K*_a_= (3.5 ± 0.2) × 10^3^ M^−1^
**15** 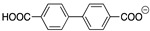	Threaded through Cavity	2:3	[H] + [G] $\overset{K_{1}}{⇄}$ [H–G] 2[H–G] + [G] $\overset{K_{2}}{⇄}$ [H_2_–G_3_]	*K*_1_ = (1.5 ± 0.1) × 10^3^ M^−1^ *K*_2_ = (1.8 ± 0.2) × 10^7^ M^−2^
**16** 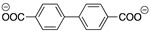	Threaded through Cavity	2:3	[H] + [G] $\overset{K_{1}}{⇄}$ [H–G] 2[H–G] + [G] $\overset{K_{2}}{⇄}$ [H_2_–G_3_]	*K*_1_ = (1.8 ± 0.2) × 10^3^ M^−1^ *K*_2_ = (1.1 ± 0.1) × 10^7^ M^−2^
**17** 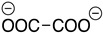	Outside Cavity	1:1	[H] + [G] $\overset{K_{a}}{⇄}$ [H–G]	*K*_a_= (1.7 ± 0.1) × 10^3^ M^−1^
**18** 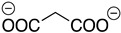	Outside Cavity	1:1	[H] + [G] $\overset{K_{a}}{⇄}$ [H–G]	*K*_a_= (3.6 ± 0.2) × 10^3^ M^−1^
**19** 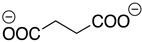	Outside Cavity	1:1	[H] + [G] $\overset{K_{a}}{⇄}$ [H–G]	*K*_a_= (4.8 ± 0.2) × 10^3^ M^−1^
**20** 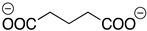	Outside Cavity	1:1	[H] + [G] $\overset{K_{a}}{⇄}$ [H–G]	*K*_a_= (7.1 ± 0.4) × 10^3^ M^−1^
**21** 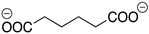	Outside Cavity	1:1	[H] + [G] $\overset{K_{a}}{⇄}$ [H–G]	*K*_a_= (1.0 ± 0.1) × 10^4^ M^−1^
**22** 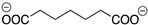	Outside Cavity	1:1	[H] + [G] $\overset{K_{a}}{⇄}$ [H–G]	*K*_a_= (2.6 ± 0.1) × 10^4^ M^−1^
**23** 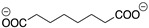	Outside Cavity	1:1	[H] + [G] $\overset{K_{a}}{⇄}$ [H–G]	*K*_a_= 4.9 ± 0.2) × 10^3^ M^−1^
**24** 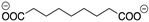	Outside Cavity	1:1	[H] + [G] $\overset{K_{a}}{⇄}$ [H–G]	*K*_a_= (4.6 ± 0.2) × 10^3^ M^−1^
**25** 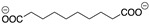	Outside Cavity	1:1	[H] + [G] $\overset{K_{a}}{⇄}$ [H–G]	*K*_a_= (6.9 ± 0.4) × 10^3^ M^−1^
**26** 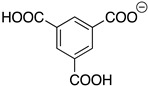	Outside Cavity	1:2	[H] + [G] $\overset{K_{1}}{⇄}$ [H–G] [H–G] + [G] $\overset{K_{2}}{⇄}$ [H–G_2_]	*K*_1_ = (3.4 ± 0.3) × 10^3^ M^−1^ *K*_2_ = (2.5 ± 0.2) × 10^3^ M^−1^
**27** 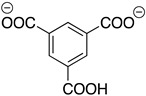	Outside Cavity	1:1	[H] + [G] $\overset{K_{a}}{⇄}$ [H–G]	*K*_a_= (3.4 ± 0.1) × 10^3^ M^−1^
**28** 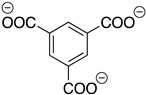	Outside Cavity	2:3	[H] + [G] $\overset{K_{1}}{⇄}$ [H–G] 2[H–G] + [G] $\overset{K_{2}}{⇄}$ [H_2_–G_3_]	*K*_1_ = (6.5 ± 0.1) × 10^3^ M^−1^ *K*_2_ = (8.5 ± 0.2) × 10^3^ M^−2^
**29** 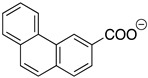	Sandwich	2:3	[H] + [G] $\overset{K_{1}}{⇄}$ [H–G] [H–G] + [G] $\overset{K_{2}}{⇄}$ [H–G_2_] [H–G] + [H–G_2_] $\overset{K_{3}}{⇄}$ [H_2_–G_3_]	*K*_1_ = (4.3 ± 0.1) × 10^3^ M^−1^ *K*_2_ = (2.9 ± 0.2) × 10^3^ M^−2^ *K*_3_ = (2.5 ± 0.2) × 10^3^ M^−2^
**30** 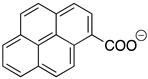	Sandwich	2:3	[H] + [G] $\overset{K_{1}}{⇄}$ [H–G] [H–G] + [G] $\overset{K_{2}}{⇄}$ [H–G_2_] [H–G] + [H–G_2_] $\overset{K_{3}}{⇄}$ [H_2_–G_3_]	*K*_1_ = (5.1 ± 0.2) × 10^3^ M^−1^ *K*_2_ = (3.2 ± 0.3) × 10^3^ M^−2^ *K*_3_ = (3.2 ± 0.2) × 10^3^ M^−2^
**31** 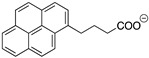	Sandwich	2:3	[H] + [G] $\overset{K_{1}}{⇄}$ [H–G] [H–G] + [G] $\overset{K_{2}}{⇄}$ [H · G_2_] [H–G] + [H–G_2_] $\overset{K_{3}}{⇄}$ [H_2_–G_3_]	*K*_1_ = (4.3 ± 0.1) × 10^3^ M^−1^ *K*_2_ = (3.0 ± 0.3) × 10^3^ M^−2^ *K*_3_ = (3.6 ± 0.1) × 10^3^ M^−2^

## Data Availability

Not applicable to this article.
